# Prognostic Potential of Cyclin D1 Expression in Colorectal Cancer

**DOI:** 10.3390/jcm12020572

**Published:** 2023-01-10

**Authors:** Sun-Young Jun, Jiyoung Kim, Nara Yoon, Lee-So Maeng, Jae Ho Byun

**Affiliations:** 1 Department of Pathology, Incheon St. Mary’s Hospital, College of Medicine, The Catholic University of Korea, Incheon 21431, Republic of Korea; 2 Division of Oncology, Department of Internal Medicine, Incheon St. Mary’s Hospital, College of Medicine, The Catholic University of Korea, Incheon 21431, Republic of Korea

**Keywords:** colon, adenocarcinoma, cyclin D1, immunohistochemistry, survival

## Abstract

Cyclin D1 is mainly known as an oncogenic driver in cancers, and the dysregulated cyclin D1/cyclin-dependent kinase (CDK) 4/6 axis is considered an attractive target for cancer therapy. Recent studies have reported that tumors respond to therapeutic interventions targeting altered cyclin D1 expression via application of the CDK4/6 inhibitor. However, the prognostic and therapeutic contributions of cyclin D1 to colorectal cancer (CRC) remain controversial. Herein, we assessed the associations between cyclin D1 expression and clinicopathological factors, including patients’ overall survival (OS) and recurrence-free survival (RFS), in 495 surgically resected primary CRCs. We also examined previous studies for cyclin D1 in CRCs. High expressions of cyclin D1 (cyclin D1^High^) was observed in 389 CRC cases (78.6%). Cyclin D1^High^ consistently predicted better patient OS and RFS in CRCs. Based on multivariate analysis, cyclin D1^High^ and young age of patients remained as independent prognosticators of higher OS rate, whereas cyclin D1^High^, females, chemotherapy, absence of nodal metastasis, and lower T-category remained as independent prognosticators of better RFS. Cyclin D1 is commonly overexpressed in CRCs, and its expression can be used as a favorable prognostic indicator in patients with CRCs; this may be important for predicting responses to subsequent CDK4/6 inhibitors.

## 1. Introduction

Cyclin D1 is a 36-kDa protein encoded by *CCND1*, which is located on chromosome 11q13 [1]; it is primarily known as a regulator of cell cycle progression and modulates the transition from G1 to S phases through interactions with cyclin-dependent kinase (CDK) 4 and CDK6 [2]. The cyclin D1/CDK pathway initiates phosphorylation of the retinoblastoma protein and relieves histone deacetylase-binding, thus enabling transcriptional activation of the S-phase genes [2]. The dysregulation of cyclin D1 transcription as well as assembly and hyperactivation of its cognate CDK result in uncontrolled cell growth, so cyclin D1 has been regarded as an oncogenic driver in cancers [3]. In addition to its well-known role in cell cycle control, cyclin D1 has been described as having other functions, including initiation of mitochondrial metabolism, regulation of gene transcription, and control of DNA damage through repairing complexes and upregulation of noncoding sequences [4,5,6]. The role of cyclin D1 in cancer initiation and progression appears to be complex and multifaceted, and its contribution to carcinogenesis remains unknown.

The high frequency of the altered cyclin D1-CDK4/6 axis in cancer has rendered it as an attractive target for cancer therapy [7]. However, cyclin D1 lacks enzymatic activity, meaning that its catalytic partners CDK4/6 can be targeted [7]. CDK4/6 inhibitors are currently being used actively in preclinical studies for cancer treatment [8]. The most commonly used CDK4/6 inhibitors—abemaciclib, palbociclib, and ribociclib—have been proven effective in the treatment of breast cancers [8]. In addition, various other cancers, including pancreatic ductal adenocarcinoma, non-small cell lung cancer (NSCLC), and melanoma, frequently show cyclin D1 overexpression and amplification, which emphasizes the potential use of CDK4/6 inhibitors in their treatment [9,10]. In colorectal carcinomas (CRCs), the therapeutic potential of CDK4/6 inhibitors has been evaluated in combination with other drugs, such as immune checkpoint, Raf, and mitogen-activated protein kinase (MAPK) inhibitors [8]. Interestingly, *KRAS*-mutant CRCs have been shown to be particularly sensitive to a combination of MAPK and CDK4/6 inhibitors [11].

Globally, CRC is the third most common type of cancer and fourth most common cause of cancer-related deaths. It was estimated that approximately 151,030 new cases of CRCs would occur in the United States in 2022 and that about 52,580 patients would die from it [12]. The incidence of CRCs in Korea is lower than in the western countries, and about 28,111 new cases of CRCs were diagnosed in Korea in 2017 [13]. Previous immunohistochemical studies on cyclin D1 in CRCs have described its prognostic significance, but conflicting results have been reported thus far [14,15]. Variations in the country of study, methodology, disease stages, population sizes, and cutoff values of cyclin D1 expression may have led to the heterogeneity in the previous studies. Most of these studies were published before 2013 and used either the Duke staging system or older versions of the tumor node metastasis (TNM) system (Table 1) [16,17,18,19,20,21,22,23,24,25,26,27,28,29,30,31,32,33,34,35]. To identify the indicators for treatment using CDK4/6 inhibitors in CRC patients, it is necessary to analyze previous studies on cyclin D1 expression in detail. It is also necessary to evaluate the associations between cyclin D1 expression and clinicopathological factors, including the recently updated TNM system and *KRAS* mutations.

In the present study, we assess the expression of cyclin D1 in both normal colonic mucosa and tumor cells of CRCs, in addition to the clinicopathological and prognostic significance of cyclin D1 expression in CRCs. We also extensively analyze previous studies examining cyclin D1 expression to predict the survival of CRC patients and compare them with our results.

## 2. Materials and Methods

### 2.1. Patients and Tissue Samples

We collected 513 patients with CRCs who underwent curative surgery between 2008 and 2012 at Incheon St. Mary’s Hospital, Republic of Korea, using our electronic database. All tumors in which the epicenter was within the colorectum were included. Of these, 10 cases without available tissue blocks and 8 cases with previous history of a cancer other than CRC and chemo- or radiotherapy were excluded. Thus, a total cohort consisting of 495 cases of surgically resected primary CRCs was finally enrolled in this study.

The clinical data included patient sex, age, operation date, additional prior or current treatment modalities such as chemotherapy and/or radiation therapy, most recent follow-up date, recurrence date, and survival status. The pathological data obtained from gross and microscopic examinations included the size, growth pattern, histological subtype, and differentiation of the tumor, along with the T and N categories, stage grouping, marginal status, nodal metastasis, as well as perineural and lymphovascular invasion. The T and N categories and stage grouping were evaluated according to the 8th American Joint Committee on Cancer (AJCC) staging system [36], and the tumor grades were based on the 5th edition of the World Health Organization (WHO) classification [37]. The study was conducted in accordance with the Declaration of Helsinki, and approved by the Institutional Review Board of Incheon St. Mary’s Hospital of the Catholic University of Korea (OC22SISI0068 and OC15TISI0050). The requirement for patient consent was waived as the data used in this study were retrospectively obtained and anonymized.

### 2.2. Immunohistochemical Analysis

The tissue microarrays (TMAs) were constructed using formalin-fixed paraffin-embedded tissue blocks. Two cores of each tumor and one core of paired normal colonic mucosa (when available) were sampled from the representative areas using a 2.0-mm punch. Then, 4-µm-thick slides were cut from the TMA blocks for immunohistochemistry (IHC). Cyclin D1 (clone SP4; Cell Marque, Darmstadt, Germany) IHC was performed using a Ventana BenchMark XT immunostainer (Ventana Medical System, Tucson, AZ, USA) according to manufacturer protocols. Nuclear immunostaining for cyclin D1 was then evaluated as the percentage and intensity of positive epithelial cells. The percentage of staining was graded on a scale of 0 to 10, with 0 (no staining in the epithelial cells), 1 (1–10%), 2 (11–20%), 3 (21–30%), and so on in 10% increments up to 10 (91–100%). The intensity of staining was graded as follows: 0, no staining; 1, weak staining; 2, moderate staining; and 3, strong staining. The immunostaining scores were calculated by multiplying the grade for the percentage of cells stained (0–10) with the grade for staining intensity (0–3), thus yielding scores ranging from 0 to 30. The final composite scores were generated from the average score for each case.

### 2.3. Molecular Analysis

Genomic DNA was extracted from the formalin-fixed paraffin-embedded tissue blocks using a QIAmp DNA Mini Kit (Qiagen Inc., Valencia, CA, USA). Mutations in codons 12 and 13 of the *KRAS* exon 1 and codon 600 of the *BRAF* exon 15 were identified using the PNA Clamp™ KRAS and BRAF Mutation Detection Kit (Panagene Inc., Daejeon, Republic of Korea) according to manufacturer protocols. The PNA Clamp™ Mutation Detection Kit is based on the peptide nucleic acid (PNA)-mediated real-time polymerase chain reaction (PCR) clamping technology [38]. The PNA is characterized by a stronger bond between the PNA/DNA than that between DNA/DNA because it lacks a charged phosphate group and does not induce electrostatic repulsion [39]. In brief, PCR amplification was performed in a final volume of 20 μL containing the template DNA, primers, PNA probe, and a SYBR Green PCR master mix. The PCR efficiency was determined by measuring the cycle threshold (Ct) value: Ct values for the control and mutation assays were obtained from the SYBR Green amplification plots, and the delta Ct (ΔCt) value was calculated by subtracting the Ct values of the tested samples from the standard Ct value of a clamping control sample. When the value of ΔCt-1 was greater than 2, the sample was considered to have mutant DNA [38].

### 2.4. Statistical Analysis

SPSS Statistics for Windows (version 28.0; IBM, Armonk, NY, USA) was used for the statistical analysis. A comparison of means was performed with the unpaired Student *t*-test, and the categorical variables were evaluated using χ^2^ and/or Fisher’s exact test. Survival curves were plotted using the Kaplan–Meier method, and comparisons of the survival rates in relation to various clinicopathological factors were conducted in terms of the log-rank test. The significance of the prognostic factors was investigated using the Cox proportional hazards model. The overall survival (OS) and recurrence-free survival (RFS) were estimated from the date of surgery to the date of event (death or last follow-up of the patient in OS; recurrence of cancer in RFS). Receiver operating characteristic (ROC) curves were generated to calculate the area under the curve (AUC), which measures the predictive power of cyclin D1 expression for accurately classifying CRC cases associated with patient survival. A *p* value of <0.05 was considered to be statistically significant.

## 3. Results

### 3.1. Clinicopathological Characteristics

There were no underlying diseases in the patients, such as inflammatory bowel dis-ease, or hereditary cancer syndromes, including familial adenomatous polyposis, Lynch syndrome, Peutz–Jeghers syndrome, and Cowden syndrome. The median OS and RFS of the patients were 41.2 and 36.3 months (range: 1.0–92.1 months for OS, 1.0–87.0 months for RFS), respectively. During follow-up, 118 patients (23.8%) showed cancer recurrence, and the median time to recurrence was 36.3 months. The baseline clinicopathological characteristics are summarized in Table 2. The patients’ ages ranged from 27 to 97 years (mean: 63.5 ± 12.4 years) and the male to female ratio was 1.5. The tumor sizes ranged from 0.7 to 14.0 cm (mean: 5.3 ± 2.4 cm). The tumor growth patterns were investigated in 471 cases, and included a polypoid pattern in 33 cases (7.0%), ulcerofungating pattern in 231 cases (49.0%), and ulceroinfiltrative pattern in 207 cases (44.0%). Most of the tumors (471, 95.2%) were of low grade. Lymphovascular invasion was observed in 197 cases (39.8%), and perineural invasion was seen in 176 cases (35.6%). Resection margins were involved in cancer in 35 cases (7.1%). According to the 8th AJCC staging system, four cases were categorized as T1 (0.8%), 25 as T2 (5.1%), 400 as T3 (80.8%), and 66 as T4 (13.3%) tumors. Nodal metastases were observed in 251 cases (50.7%), comprising 144 N1 (29.1%) and 107 N2 (21.6%). Distant metastases were identified in two cases (0.4%); one each of peritoneal and bony metastases. Consequently, the tumors were classified into the following stages: I (5, 1.0%), II (237, 47.9%), III (251, 50.7%), and IV (2, 0.4%). Chemotherapy and radiation therapy were performed in 375 (75.8%) and 58 (11.7%) cases, respectively. The status of *KRAS* and *BRAF* was respectively assessed in 242 and 206 patients. Of these, *KRAS* mutations were observed in 38.4% (93/242) of tumors, while *BRAF* mutations were found in 6.3% (13/206) of tumors.

### 3.2. Cyclin D1 Expression

**Figure 1:**
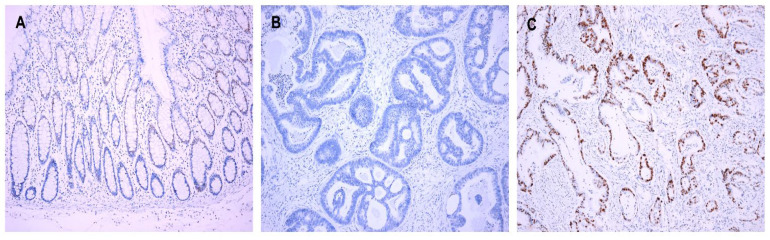
Cyclin D1 expression. (**A**) Expression in the transitional zone of the crypt at the lower part of the gland in the normal mucosa. (**B**) Cyclin D1^Low^ and (**C**) Cyclin D1^High^ in CRCs. Original magnification ×100.

There were interpretable cores of the immunostained TMAs of cyclin D1 in 422 cases of normal colonic mucosa. In 14.7% (62/422) of the normal mucosa cases, cyclin D1 was expressed in the transitional zone of the crypt at the lower part of the gland (Figure 1A); of these 62 cases, most (54/62, 87.1%) showed weak staining intensity of cyclin D1, whereas eight cases (8/62, 12.9%) displayed moderate intensity. The final staining scores of cyclin D1 in normal colonic epithelium ranged from 1 to 5 in the following descending order: 47 cases (75.8%) with score 1; 11 cases (17.7%) with score 2; two cases (3.3%) with score 4; one case (1.6%) with score 3; one case with score 5 (1.6%). In the CRCs, cyclin D1 was expressed in 86.7% (429/495) of the cases. The staining scores ranged from 1 to 30, and most of them (389/429, 90.6%) showed a staining score of 1 or higher. The staining scores of cyclin D1 expression were higher in CRCs than in normal colonic mucosa (mean: 4.5 ± 5.4 vs. 0.2 ± 0.6; *p* < 0.001). Based on ROC curve analysis to maximize the sensitivity and specificity of cyclin D1 expression in predicting CRC patients’ survival, staining scores ≥ 1 were defined as high expression (Cyclin D1^High^). Cyclin D1^High^ was observed more often in CRCs than in normal colonic mucosa (*p* < 0.001). The clinicopathological correlations of cyclin D1 expression are summarized in Table 2. Cyclin D1^High^ was observed in 389 out of the 495 cases (78.6%) (Figure 1B,C). Cyclin D1^High^ was found to be significantly related to younger age (*p* = 0.037) and female sex (*p* = 0.014). Neither *KRAS* nor *BRAF* was associated with cyclin D1 expression.

### 3.3. Prognostic Significance of Cyclin D1 Expression

**Figure 2:**
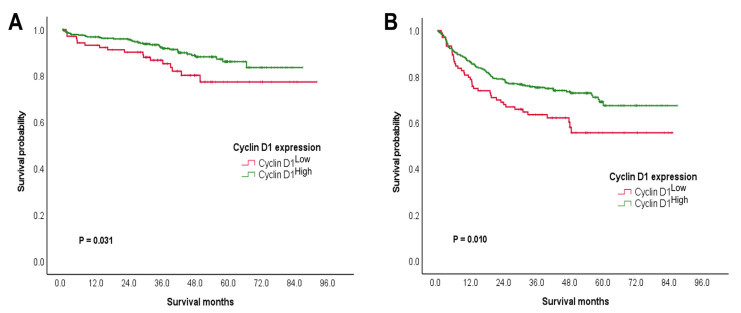
Prognostic stratification of cyclin D1 expression. Favorable prognostic potential of cyclin D1^High^ to predict (**A**) OS and (**B**) RFS.

CRCs with cyclin D1^High^ were found to be related to improved OS and RFS among the patients (Table 3 and Table 4). In a univariate analysis for the OS, CRC patients with cyclin D1^High^ had significantly higher 5-year survival rates (5-YSRs) than those with cyclin D1^Low^ (86.2% vs. 77.4%, *p* = 0.031; Figure 2A). Further, younger age of the patient (*p* = 0.013) and chemotherapy (*p* = 0.014) were both associated with better OS. Following a multivariate analysis, cyclin D1^High^ (*p* = 0.045) and younger age of the patient (*p* = 0.016) were both found to be independent prognostic indicators for improved OS.

A univariate analysis for RFS revealed that CRC patients with cyclin D1^High^ had significantly higher 5-YSRs than those with cyclin D1^Low^ (69.1% vs. 55.7%, *p =* 0.010; Figure 2B). Moreover, female patients (*p* = 0.014), low grade of tumor (*p* = 0.017), clear resection margin (*p* = 0.011), chemotherapy (*p* = 0.043), absence of nodal metastasis (*p* = 0.021), lower T category (*p* < 0.001), and lower stage grouping (*p* = 0.009) were all associated with better RFS. Based on a multivariate analysis, cyclin D1^High^ (*p* = 0.046), female sex (*p* = 0.004), chemotherapy (*p* = 0.011), absence of nodal metastasis (*p* = 0.041), and lower T category (*p* < 0.001) were found to be independent prognostic indicators for better RFS.

## 4. Discussion

We assessed cyclin D1 expression in a large cohort of patients with CRCs (*n* = 495) and identified higher cyclin D1 expression in CRC than in normal colonic mucosa. Further, cyclin D1^High^ was consistently found to be a favorable prognostic marker for predicting patients’ OS and RFS.

Although cyclin D1 has been regarded as an oncogenic driver in cancers, the prognostic effects of cyclin D1 have been inconclusive thus far, as no consensus has been reached. The associations between cyclin D1 and good prognosis have not been uncommonly described in NSCLC, breast cancer, and bladder cancer [15,40,41,42]. In CRCs, the prognostic impact of cyclin D1 expression has been inconsistently described in 21 case-controlled studies, including the present study (Table 1); six studies reported cyclin D1^High^ to be a favorable prognostic factor [16,17,20,23,28], six studies reported that it was unfavorable [24,25,26,27,34], and nine studies reported that there was no association [18,19,21,22,30,31,32,33,35]. In addition, two meta-analysis studies demonstrated different results for the prognostic impact of cyclin D1 in CRCs (Table 1); Li et al. proposed cyclin D1 as an unfavorable prognostic factor by analyzing 22 studies [14], while Binabaj et al. found no significant prognostic effects of cyclin D1 from an analysis of 15 studies [15]. Some limitations existed in these two meta-analyses, including variable staging methods and lengths of follow-up as well as inconsistently defined cutoff values with publication bias. Based on an analysis of previous studies on CRCs, it was assumed that differences in the sample sizes, clone used, tissue sections, and cutoff points could affect the prognostic assessment of cyclin D1 expression.

We analyzed the prognostic impact of cyclin D1^High^ from previous studies in terms of the sample size. Of the eight studies with relatively large cohorts of CRCs containing ≥200 cases, one half (4/8) described favorable effects of cyclin D1^High^ whereas the other half did not find it to have any prognostic significance. On the other hand, among 12 studies on <200 CRC cases each, seven found a prognostic impact of cyclin D1^High^, and most of these (6/7, 85.7%) showed unfavorable effects. Therefore, differences in the sample sizes might contribute to evaluation of the prognosticity of cyclin D1 expression. The frequency of cyclin D1 expression among CRCs has been found to range from 8.0% to 75.0% in previous studies [16,17,18,19,20,21,22,23,24,25,26,27,28,30,31,32,33,34,35]. Studies from eastern countries with homogeneous ethnic groups—such as Korea and Japan—have shown wide ranges of frequencies of cyclin D1 expression (13.9% to 72.5%) [23,24,25], and other studies with heterogeneous ethnic groups, such as studies from the USA, have reported various frequencies of cyclin D1 expression (15.0% to 54.8%) [20,31]. Therefore, the various cyclin D1 expression frequencies found in previous studies may not be related to the diverse ethnic backgrounds. Interestingly, when limited to studies including ≥200 cases each, cyclin D1^High^ was identified as a good prognostic factor in studies conducted in both Korea and the USA [20,23].

We analyzed the prognostic impact of cyclin D1^High^ from previous studies with regard to the study method. Several techniques, such as the clone used (SP4, P2D11F11, and DCS-6, etc.) and tissue section type (conventional section vs. TMA), were applied for cyclin D1 IHC. Comparing TMA with whole-section histology, 2–4 tissue cores of TMAs are representative with a concordance rate of 95–97%, so numerous IHC studies using TMA have been performed [43]. Torlakovic et al. compared the anti-cyclin D1 antibodies, including monoclonal SP4, P2D11F11, and DCS-6 as well as polyclonal CP236 and 06-137, in malignant lymphomas [44]. Among these antibodies, SP4 was found to produce the strongest staining with a high sensitivity of 95%, and it is thus considered to be suitable for the optimal detection of cyclin D1 expression [44]. Most previous studies (15/20, 75.0%) on CRCs reported cyclin D1 IHC on conventionally sectioned slides, whereas only a few (5/20, 25.0%) were based on the TMA [16,17,18,19,20,21,22,23,24,25,26,27,28,30,31,32,33,34,35]. Of the five studies that used TMAs, 40.0% (2/5) investigated cyclin D1 expression with SP4 [20,23], and 80.0% (4/5) identified a favorable prognostic effect of cyclin D1^High^. Meanwhile, in studies using various clones (e.g., P2D11F11 and DCS-6) and conventionally sectioned slides for IHC, only two studies by McKay et al. and Holland et al. (2/15, 13.3%) reported favorable prognosticity of cyclin D1^High^ [16,28]. Interestingly, studies using both TMA and SP4 found cyclin D1^High^ to be a good prognostic factor for cyclin D1 in CRCs [20,23]. Similarly, we collected 495 CRC cases, selected a clone of SP4, constructed the TMA for cyclin D1 IHC, and found a favorable prognostic effect of cyclin D1^High^.

Previous studies on cyclin D1 expression in CRCs have applied various cutoff points for cyclin D1^High^ without providing detailed descriptions [16,17,18,19,20,21,22,23,24,25,26,27,28,29,30,31,32,33,34,35]. In the present study, we set an objective cutoff value based on ROC analysis. We dichotomized cyclin D1 expression based on the optimized cutoff determined in terms of its association with OS. When using a cutoff point for a staining score ≥ 1, the AUC was 0.53, indicating poor accuracy in the ability to discriminate patient survival, with a sensitivity of 32.7% and specificity of 80.0%. In previous works, cytoplasmic staining of cyclin D1 has been demonstrated [17,26,28], which has been shown to be associated with improved OS [28]. In the study by Torlakovic et al., cytoplasmic reactions were found with DCS-6, 06-137, and P2D1F11, while SP4 and CP236 did not produce cytoplasmic staining [44]. We used SP4 in this study, and no cytoplasmic staining of cyclin D1 was found.

The mechanism of cyclin D1 activity in cancer appears to be complex. Cyclin D1 overexpression increases stem cell-like behaviors and migrations in estrogen receptor (ER)-positive breast cancer, while the opposite is seen in ER-negative cells, thus reflecting the fundamentally different effects of cyclin D1 expression in ER-positive and ER-negative breast cancers [41]. Further, the distinctly different prognostic roles of cyclin D1 in superficial and muscle-invasive bladder cancers reinforce the concept that urothelial bladder cancer comprises two different diseases with distinct underlying molecular mechanisms [42]. Ogino et al. similarly proposed the existence of molecular differences between CRCs with cyclin D1^High^ and cyclin D1^Low^; CRCs with cyclin D1^Low^ may have bypassed cyclin D1 activation, resulting in more aggressive behavior than cyclin D1^High^ tumors through the accumulation of multiple genetic and epigenetic events during colorectal carcinogenesis [20]. There is evidence for the alternative roles of cyclin D1 in neoplastic cells, including involvement in apoptosis and growth suppression, which may account for its beneficial effect on prognosis [45,46]. Thus, further studies are needed to understand the mechanism of the favorable prognostic effects of cyclin D1 in malignancies.

In a recent study examining cyclin D1 in breast cancer, the amplification of *CCND1* was associated with increased risk of disease recurrence, whereas a higher expression of cyclin D1 protein was associated with decreased recurrence risk [47]. In our study, cyclin D1^High^ was found to be significantly related to a lower recurrence rate (*p* = 0.029, data not shown) and longer RFS among CRC patients. In NSCLC, cooperation between cyclin D1 and *KRAS* was suggested though the involvement of the activated extracellular signal-regulated kinase (ERK)/cyclin D1 pathway in *KRAS*-driven lung tumorigenesis [48]. However, we did not find any relationship between *KRAS* mutation and cyclin D1 expression in CRCs. It has recently been suggested that *CCND1* amplification may be associated with poor response to antiestrogen therapy and that ribociclib and abemaciclib may contribute to increased response rates when combined with endocrine therapy in hormone-responsive advanced breast cancer [49]. Nonetheless, we did not find any associations between cyclin D1 expression and chemotherapy in CRCs. Further studies are thus needed to understand the pivotal role of cyclin D1 in shaping the development of the tumor microenvironment and in its therapeutic efficacy for successful clinical translation to treating patients with cancer.

A few previous studies have investigated cyclin D1 expression in normal colonic mucosa [29,50,51]. In a study by Arber et al., cyclin D1 was expressed in 30% (8/27) of CRCs and 34% (12/35) of adenomatous polyps but not in normal mucosa (*n* = 23). The normal mucosal tissues were obtained from resection margins of CRCs (*n* = 13) and colonoscopic biopsy specimens from healthy individuals (*n* = 10) [50]. Bahnassy et al. also demonstrated that there was no expression of cyclin D1 in normal colonic tissues obtained from autopsy specimens (*n* = 20) [29]. Khor and colleagues investigated cyclin D1 expression in both CRCs and apparently normal adjacent colonic tissues; the immunoreactivity of cyclin D1 was found in 10.6% (5/47) of CRCs, but no reactions were detected in apparently normal adjacent colonic mucosa [51]. In the present study, cyclin D1 was expressed in 14.7% (62/422) of normal colonic mucosa adjacent to the CRC, which was a higher frequency than expected. Cyclin D1 was expressed in the transitional zone of the crypt at the lower part of the gland, most of which showed a staining score of 1. However, it is difficult to compare our results because extant studies have not described the intensity and distribution of cyclin D1 immunoreactivity in normal colonic mucosa [29,50,51]. Experimental studies by Pysz et al. demonstrated the differential regulation of cyclin D1 expression by the protein kinase C (PKC) family in intestinal homeostasis and tumorigenesis [52]. PKCα acts as a tumor suppressor in intestinal epithelial cells via downregulation of cyclin D1, while PKCε appears to function as an oncogene in both transformed IEC-18 and colon cancer cells via positive regulation of cyclin D1 [52,53]. PKCε activity is localized to proliferate in the intestinal crypt cells, which coincides with the expression of cyclin D1 in these cells [52]. Although PKCα tends to be lost in intestinal tumorigenesis, PKCε is retained in the CRC cells as a physiologically relevant regulator of cyclin D1 [52]. In this study, cyclin D1 was expressed in the crypt cells of normal colonic mucosa and was more frequently expressed in the tumor cells of CRCs than in normal colonic mucosa, corresponding to the findings of Pysz et al. [52]. However, further studies are needed to define the contributions of cyclin D1 in the tumorigenesis of CRCs.

## 5. Conclusions

The present study, which is one of the largest clinicopathological studies on CRCs, elucidates the characteristics of cyclin D1 and explores its prognostic significance for both OS and RFS. We determined cyclin D1^High^ as an independent predictor through a multivariate analysis. Knowledge of the expression patterns of cyclin D1 may be important for predicting responses to subsequent chemotherapy regimens, including those using CDK4/6 inhibitors. Furthermore, the use of CDK inhibitors, either as single agents or in combination with other drugs, may be a useful therapeutic strategy in the development of new treatments for CRCs.

## Figures and Tables

**Table 1:** Previous studies of cyclin D1 expression in CRC.

Study	Country (yr)	IHC	*N*	Clone Used	Cyclin D1^High^		Outcome	
					Cut-Off Level	*N* (%)	Stage	Survival
*N* ≥ 200								
Present	Korea	TMA	495	SP4	Score ≥ 1	389 (78.6)	TNM	Better OS and RFS
McKay [16]	UK (2002)	Whole section	249	P2D11F11	>5%	137 (55.0)	Duke	Better OS
Hilska [17]	Finland (2005)	TMA	363	P2D11F11	≥1%	99 (27.3)	Duke	Better OS
Bondi [18]	Norway (2005)	Whole section	219	NS	≥5%	24 (11.0)	Duke	Not related to OS
Von Stockmar–Von Wangenheim [19]	Germany (2008)	Whole section	200	P2D11F11	>5%	150 (75.0)	TNM	Not related to OS
Ogino [20]	USA (2009)	TMA	602	SP4	>50%	330 (54.8)	TNM	Better CSS
Fang [21]	China (2009)	TMA	532	NS	≥10%	380 (71.4)	TNM	Not related to OS
Belt [22]	Netherlands (2012)	TMA	379	NS	Score ≥ 8	211 (55.7)	TNM	Not related to OS
Jang [23]	Korea (2012)	TMA	217	SP4	≥30%	158 (72.5)	TNM	Better OS and RFS
Li [14]	China (2014)	Meta-analysis	22 studies	−	−	−	−	Worse OS and DFS
Binabaj [15]	Iran (2020)	Meta-analysis	15 studies	−	−	−	−	Not related to OS
*N* < 200								
Maeda [24]	Japan (1997)	Whole section	101	NS	>50%	14 (13.9)	TNM	Worse DFS
Maeda [25]	Japan (1998)	Whole section	123	NS	>50%	20 (16.3)	TNM	Worse OS and DFS
McKay [26]	UK (2000)	Whole section	100	P2D11F11	>50%	8 (8.0)	Duke	Worse OS
Bhatavdekar [27]	India (2001)	Whole section	98	P2D11F11	≥1%	30 (30.6)	Duke	Worse OS
Holland [28]	UK (2001)	Whole section	126	DCS-6	>10%	74 (58.7) *	Duke	Better OS
Bahnassy [29]	Egypt (2004)	Whole section	60	DCS-6	Index ≥ 6.1	41 (68.3)	TNM	Worse OS
Bondi [30]	Norway (2004)	Whole section	162	NS	>5%	NS	Duke	Not related to OS
Moore [31]	USA (2004)	Whole section	40	DCS-6	>10%	6 (15.0)	TNM	Not related to RFS
Kouraklis [32]	Greece (2006)	Whole section	111	NS	>5%	71 (63.9)	Duke	Not related to OS **
Lyall [33]	UK (2006)	Whole section	90	P2D11F11	>5%	46 (51.1)	TNM	Not related to OS
Mao [34]	China (2011)	Whole section	169	NS	>5%	95 (56.2)	TNM	Worse OS
Tsai [35]	Taiwan (2013)	Whole section	100	NS	Score ≥ 2	49 (49.0)	TNM	Not related to OS and DFS

Abbreviations: CRC, colorectal carcinoma; IHC, immunohistochemical; TMA, tissue microarray; OS, overall survival; RFS, recurrence-free survival; CSS, cancer-specific survival; NS, not stated; DFS, disease-free survival. * Cyclin D1 was expressed in the nucleus in 22 tumors and expressed exclusively in the cytoplasm in 52 cases. ** Patients with advanced Duke stage and those less than 70 years old both had worse OS.

**Table 2:** Association between cyclin D1 expression and clinicopathological factors in 495 patients with CRC.

Clinicopathological Factors, *N* (%)		Total	Cyclin D1^Low^	Cyclin D1^High^	*p*
			106 (21.4)	389 (78.6)	
Age (yr, mean ± SD)			65.6 ± 11.1	62.9 ± 12.7	0.037 *
Age (yr)	≤50	78 (15.8)	12 (15.4)	66 (84.6)	0.178
	>50	417 (84.2)	94 (22.5)	323 (77.5)	
Sex	Male	293 (59.2)	74 (25.3)	219 (74.7)	0.014 *
	Female	202 (40.8)	32 (15.8)	170 (84.2)	
Tumor size (cm, mean ± SD)			5.3 ± 2.4	5.3 ± 2.4	0.921
Growth pattern (*n* = 471) ^§^	Polypoid	33 (7.0)	10 (30.3)	23 (69.7)	0.450
	Ulcerofungating	231 (49.0)	47 (20.3)	184 (79.7)	
	Ulceroinfiltrative	207 (44.0)	43 (20.8)	164 (79.2)	
Differentiation	Low grade	471 (95.2)	99 (21.0)	372 (79.0)	0.442
	High grade	24 (4.8)	7 (29.2)	17 (70.8)	
Lymphovascular invasion	Absent	298 (60.2)	64 (21.5)	234 (78.5)	1.000
	Present	197 (39.8)	42 (21.3)	155 (78.7)	
Perineural invasion	Absent	319 (64.4)	62 (19.4)	257 (80.6)	0.138
	Present	176 (35.6)	45 (25.1)	131 (74.9)	
Margin status	No involvement	460 (92.9)	97 (21.1)	363 (78.9)	0.523
	Involved by cancer	35 (7.1)	9 (25.7)	26 (74.3)	
Chemotherapy	Absent	120 (24.2)	32 (26.7)	88 (73.3)	0.125
	Present	375 (75.8)	74 (19.7)	301 (80.3)	
Radiotherapy	Absent	437 (88.3)	98 (22.4)	339 (77.6)	0.172
	Present	58 (11.7)	8 (13.8)	50 (86.2)	
Nodal metastasis	Absent	244 (49.3)	48 (19.7)	196 (80.3)	0.381
	Present	251 (50.7)	58 (23.1)	193 (76.9)	
T category	T1	4 (0.8)	0	4 (100)	0.263
	T2	25 (5.1)	9 (36.0)	16 (64.0)	
	T3	400 (80.8)	83 (20.7)	317 (79.3)	
	T4	66 (13.3)	14 (21.2)	52 (78.8)	
N category	N0	244 (49.3)	48 (19.7)	196 (80.3)	0.455
	N1	144 (29.1)	36 (25.0)	108 (75.0)	
	N2	107 (21.6)	22 (20.6)	85 (79.4)	
Stage grouping	Stage I	5 (1.0)	3 (60.0)	2 (40.0)	0.069
	Stage II	237 (47.9)	45 (19.0)	192 (81.0)	
	Stage III	251 (50.7)	57 (22.7)	194 (77.3)	
	Stage IV	2 (0.4)	1 (50.0)	1 (50.0)	
*KRAS* (*n* = 242) ^§^	Absent	149 (61.6)	23 (15.4)	126 (84.6)	0.092
	Present	93 (38.4)	23 (24.7)	70 (75.3)	
*BRAF* (*n* = 206) ^§^	Absent	193 (93.7)	39 (20.2)	154 (79.8)	0.470
	Present	13 (6.3)	1 (7.7)	12 (92.3)	

Abbreviations: CRC, colorectal carcinoma. * Significant at the level *p* < 0.05. ^§^ Only calculated using cases with available information.

**Table 3:** Association between clinicopathological factors and OS in 495 patients with CRC.

Characteristics		Univariate		Multivariate	
		5-YSR (%)	*p*	HR (95% CI)	*p*
Cyclin D1 expression	Cyclin D1^Low^	77.4	0.031 *	0.561 (0.319–0.987)	0.045 *
	Cyclin D1^High^	86.2			
Age (yr) ^§^		1.031 (1.006–1.056)	0.013 *	1.030 (1.006–1.056)	0.016 *
Age (yr)	≤50	86.8	0.631		
	>50	83.7			
Sex	Male	82.0	0.059		
	Female	87.3			
Tumor size (cm) ^§^		1.041 (0.935–1.159)	0.465		
Growth pattern (*n* = 471) ^¶^	Polypoid	89.5	0.921		
	Ulcerofungating	84.7			
	Ulceroinfiltrative	84.5			
Differentiation	Low grade	84.0	0.966		
	High grade	90.6			
Lymphovascular invasion	Absent	84.9	0.573		
	Present	83.3			
Perineural invasion	Absent	84.6	0.247		
	Present	82.8			
Margin status	No involvement	84.4	0.164		
	Involved by cancer	61.6			
Chemotherapy	Absent	80.0	0.014 *	0.650 (0.353–1.198)	0.168
	Present	85.6			
Radiotherapy	Absent	84.9	0.409		
	Present	76.9			
Nodal metastasis	Absent	86.9	0.147		
	Present	81.5			
T category	T1–T3	84.8	0.314		
	T4	80.2			
N category	N0	86.9	0.159		
	N1	85.4			
	N2	75.3			
Stage grouping	Stages I–II	87.2	0.090		
	Stages III–IV	84.5			
*KRAS* (*n* = 242) ^¶^	Absent	88.9	0.132		
	Present	77.0			
*BRAF* (*n* = 206) ^¶^	Absent	85.4	0.281		
	Present	79.5			

Abbreviations: OS, overall survival; CRC, colorectal carcinoma; 5-YSR, 5-year survival rate; HR, hazard ratio; CI, confidence interval. * Significant at the level *p* < 0.05. ^§^ Displayed as a form of HR with 95% CI. ^¶^ Only calculated using cases with available information.

**Table 4:** Association between clinicopathological factors and RFS in 495 patients with CRC.

Characteristics		Univariate		Multivariate	
		5-YSR (%)	*p*	HR (95% CI)	*p*
Cyclin D1 expression	Cyclin D1^Low^	55.7	0.010 *	0.688 (0.476–0.994)	0.046 *
	Cyclin D1^High^	69.1			
Age (yr) ^§^		1.013 (0.999–1.028)	0.072		
Age (yr)	≤50				
	>50				
Sex	Male	60.0	0.014 *	0.588 (0.408–0.846)	0.004 *
	Female	76.4			
Tumor size (cm) ^§^		1.020 (0.953–1.092)	0.569		
Growth pattern (*n* = 471) ^¶^	Polypoid	72.7	0.689		
	Ulcerofungating	68.9			
	Ulceroinfiltrative	63.5			
Differentiation	Low grade	67.1	0.017 *	1.147 (0.745–2.694)	0.287
	High grade	46.2			
Lymphovascular invasion	Absent	68.2	0.123		
	Present	63.2			
Perineural invasion	Absent	67.8	0.113		
	Present	62.6			
Margin status	No involvement	68.5	0.011 *	1.679 (0.985–2.865)	0.057
	Involved by cancer	41.9			
Chemotherapy	Absent	57.5	0.043 *	0.607 (0.413–0.892)	0.011 *
	Present	68.5			
Radiotherapy	Absent	68.2	0.100		
	Present	45.0			
Nodal metastasis	Absent	73.1	0.021 *	1.445 (1.015–2.057)	0.041 *
	Present	59.3			
T category	T1-pT3	68.8	<0.001 *	2.134 (1.408–3.233)	<0.001 *
	T4	47.8			
N category	N0	73.1	0.059		
	N1	60.4			
	N2	57.5			
Stage grouping	Stages I–II	73.8	0.009 *		
	Stages III–IV	58.8			
*KRAS* (*n* = 242) ^¶^	Absent	69.0	0.313		
	Present	47.2			
*BRAF* (*n* = 206) ^¶^	Absent	59.1	0.979		
	Present	72.7			

Abbreviations: RFS, recurrence free survival; CRC, colorectal carcinoma; 5-YSR, 5-year survival rate; HR, hazard ratio; CI, confidence interval. * Significant at the level *p* < 0.05. ^§^ Displayed as a form of HR with 95% CI. ^¶^ Only calculated using cases with available information.

## Data Availability

The datasets generated during and/or analyzed during the current study are not publicly available, but are available from the corresponding author on reasonable request.
